# Endogenous microRNA triggered enzyme-free DNA logic self-assembly for amplified bioimaging and enhanced gene therapy via in situ generation of siRNAs

**DOI:** 10.1186/s12951-021-01040-x

**Published:** 2021-09-26

**Authors:** Qinghua Jiang, Shuzhen Yue, Kaixin Yu, Tian Tian, Jian Zhang, Huijun Chu, Zhumei Cui, Sai Bi

**Affiliations:** 1 Department of Obstetrics and Gynecology, The Affiliated Hospital of Qingdao University, Qingdao University, Qingdao, 266003 People’s Republic of China; 2 College of Chemistry and Chemical Engineering, Qingdao University, Qingdao, 266071 People’s Republic of China

**Keywords:** DNA nanotechnology, miRNA, siRNA, Gene therapy, Cervical carcinoma

## Abstract

**Background:**

Small interfering RNA (siRNA) has emerged as a kind of promising therapeutic agents for cancer therapy. However, the off-target effect and degradation are the main challenges for siRNAs delivery. Herein, an enzyme-free DNA amplification strategy initiated by a specific endogenous microRNA has been developed for in situ generation of siRNAs with enhanced gene therapy effect on cervical carcinoma.

**Methods:**

This strategy contains three DNA hairpins (H1, H2/PS and H3) which can be triggered by microRNA-21 (miR-21) for self-assembly of DNA nanowheels (DNWs). Notably, this system is consistent with the operation of a DNA logic circuitry containing cascaded “AND” gates with feedback mechanism. Accordingly, a versatile biosensing and bioimaging platform is fabricated for sensitive and specific analysis of miR-21 in HeLa cells via fluorescence resonance energy transfer (FRET). Meanwhile, since the vascular endothelial growth factor (VEGF) antisense and sense sequences are encoded in hairpin reactants, the performance of this DNA circuit leads to in situ assembly of VEGF siRNAs in DNWs, which can be specifically recognized and cleaved by Dicer for gene therapy of cervical carcinoma.

**Results:**

The proposed isothermal amplification approach exhibits high sensitivity for miR-21 with a detection limit of 0.25 pM and indicates excellent specificity to discriminate target miR-21 from the single-base mismatched sequence. Furthermore, this strategy achieves accurate and sensitive imaging analysis of the expression and distribution of miR-21 in different living cells. To note, compared to naked siRNAs alone, in situ siRNA generation shows a significantly enhanced gene silencing and anti-tumor effect due to the high reaction efficiency of DNA circuit and improved delivery stability of siRNAs.

**Conclusions:**

The endogenous miRNA-activated DNA circuit provides an exciting opportunity to construct a general nanoplatform for precise cancer diagnosis and efficient gene therapy, which has an important significance in clinical translation.

**Graphic abstract:**

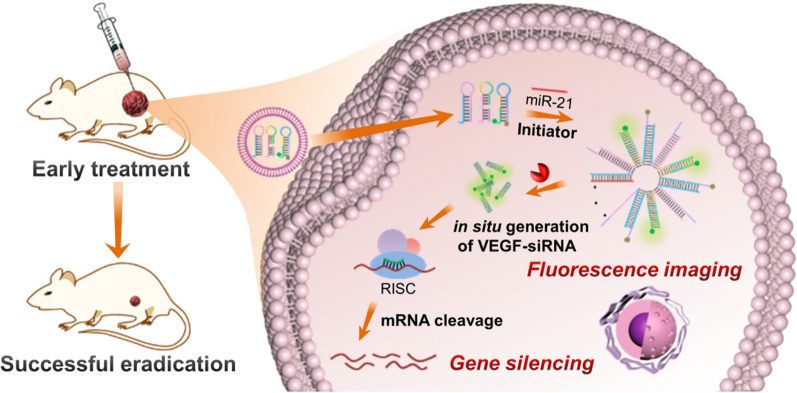

**Supplementary Information:**

The online version contains supplementary material available at 10.1186/s12951-021-01040-x.

## Introduction

SiRNAs are short double-stranded RNA molecules with 20–25 base pairs, which have acted as efficient biotherapeutic agents to treat many diseases by inducing post-transcription gene silencing [1–6]. However, the delivery of naked siRNAs by lipids, polymers and nanomaterials often shows the limitations of non-specific absorption, low efficiency of cellular uptake and potential cytotoxicity, which thus hamper their clinical applications [7–10]. Recently, DNA nanotechnology has rapidly developed to construct a variety of nanocarriers for siRNA delivery based on the good biocompatibility and programmability, such as DNA origami [11], DNA nanohydrogels [12], and so on. For these strategies, the integration of siRNAs into DNA nanostructures mainly relies on the hybridization or chemical modification, thus, the unavoidable leakage and degradation, as well as the limited payload capacity may diminish the gene therapy efficiency [13–15]. As an alternative method, in situ generation of siRNAs can significantly enhance the specificity and delivery stability, which thus provides a robust and powerful strategy for cancer therapy [16, 17].

It has been found the aberrant expression of miRNA is closely related to many cancers, such as lung carcinoma [18], breast cancer [19], pancreatic cancer [20], and so on. Therefore, it is of far reaching importance to detect miRNA for early cancer diagnosis [21, 22]. At present, northern blotting is commonly used for miRNA detection [23, 24]. Also, some enzyme-mediated nucleic acid signal amplification methods, including polymerase chain reaction (PCR) [25] and rolling circle amplification (RCA) [26], have been widely used for quantification of miRNA [27]. However, the drawbacks such as harsh reaction conditions, low sensitivity, and the involvement of exotic enzymes make these methods unsuitable for further applications in living cells [28, 29]. In recent years, DNA nanotechnology-based imaging methods have been rapidly developed for multiple bioanalysts detection [30–33]. Notably, a variety of enzyme-free DNA nanotechnology-based methods, especially DNA strand displacement reactions, have been developed for in situ imaging and quantification of miRNA in living cells due to the advantages of simple operation and isothermal features [34–39]. More importantly, as an important biomarker of cancers, the combination of biosensing and bioimaging of miRNA with therapy modalities is of great significance to monitor disease progression in response to treatment, which may offer innovative platforms for theranostics and precision medicine [40–44].

Herein, a miRNA-responsive AND-gate cascaded DNA logic circuit with target feedback function has been developed, which has achieved enzyme-free isothermal amplification of miR-21 in living cells and in situ generation of VEGF siRNA for enhanced gene therapy of cervical carcinoma. On the basis of catalytic hairpin assembly (CHA) reaction [45, 46], three kinds of DNA building blocks (H1, H2/PS and H3) are rationally designed, which can be initiated by target miRNA for the assembly of DNWs. The DNA circuit operates in a catalytic fashion with the recycling of target miRNA for signal amplification, resulting in the sensitive and selective biosensing and bioimaging of miR-21 in HeLa cells. Moreover, the VEGF antisense and sense sequences are incorporated into H2 and H3. As a result, numerous VEGF siRNAs are in situ generated in DNWs, achieving the efficient inhibition of VEGF mRNA and protein expression for gene therapy of cervical carcinoma. Compared to the traditional methods for siRNA delivery, our proposed strategy with in situ generation of siRNA in living cells presents a significantly improved delivery efficiency and stability, which thus offers a robust and powerful approach for specific gene silencing and cancer therapy. It should be noted that unlike traditional hairpin structure, H2 with a single-stranded tail (12 nt) is designed to hybridize with a protection strand (PS), in which the sequences for miRNA recognition (domains 1–2–3) and siRNA generation (domains 5–6) are independently with each other. Thus, this strategy be readily applied for the response of any miRNA and in situ generation of various siRNAs only by rationally designing the DNA hairpins according to the sequences of corresponding miRNA and siRNA, which presents the universality of the proposed DNA circuit.

## Materials and methods

### Materials and reagents

All oligonucleotides used in this work were synthesized and purified by Sangon Biotechnology Co., Ltd. (Shanghai, China), and their sequences are listed in Additional file 1: Table S1. Fetal bovine serum (FBS) and Dulbecco’s modified Eagle’s medium (DMEM) were obtained from Biological Industries (Israel) and HyClone (USA), respectively. Lipofectamine 3000 was ordered from ThermoFisher scientific (USA). Annexin V-FITC apoptosis detection kit was purchased from CoWin Biosciences (Beijing, China). Hoechst 33342 solution, 4 S Red Plus Nucleic Acid Stain and *N*,*N*,*N*′,*N*′-tetramethyl ethylenediamine (TEMED) were ordered from Sangon Biotechnology Co., Ltd. (Shanghai, China). All the reagents were of analytical grade and used without further purification.

### Preparation of DNA hairpins

The stock solutions of oligonucleotides (100 µM) were prepared using deionized ultrapure water, which were further diluted to 10 µM using TE buffer (10 mM Tris-HCl, 1 mM EDTA-2Na, 12.5 mM MgCl_2_, pH 7.4). To obtain the desirable secondary structures, the DNA hairpin strands (H1, H2 and H3) were respectively annealed in TE buffer by heating to 95 °C for 10 min, followed by cooling to 25 °C with a rate of 0.1 °C/s and standing at 25 °C for 4 h before each use. Further, H2 was incubated with equal amount of protect strand (PS) at 37 °C for 2 h to form the H2/PS hybrid.

### Native polyacrylamide gel electrophoresis

The 8% polyacrylamide gel (PAGE) was prepared according to the previous study [36], followed by adding 10 µL of each sample and 2 µL of 10× loading buffer. Then, the native PAGE was running at 170 V for 5 min and 110 V for 35 min in 1× TAE buffer (10 mM Tris, 1 M glacial acetic acid, 1 mM EDTA-2Na, 12.5 mM MgCl_2_, pH 8). Finally, the resulting gel was stained with 4S Red Plus solution for about 30 min and recorded by Tanon 2500R gel imaging system (Shanghai, China).

### Atomic force microscopy imaging

A 10 µL of each sample was deposited onto the freshly cleaved mica for 30 min. Then, the mica surfaces were washed using deionized ultrapure water for three times, followed by drying at room temperature. Finally, the samples were scanned by Being Nano-Instruments CSPM-4000 system (Guangzhou, China) under the tapping mode. The obtained images were analyzed using CSPM Console software.

### Fluorescence measurements

A 2 µL of miR-21 with different concentrations was added to the mixture of H1, H2/PS and H3 (10 µL and 200 nM for each species), respectively. The real-time fluorescence was monitored on a LineGene 9600 Real-Time detection system (Hangzhou, China) at an interval of 30 s (λ_ex_ = 470 nm and λ_em_ = 525 nm) for 4 h. The reaction temperature was set at 37 °C. The corresponding fluorescence spectra at 4 h were recorded on a F-7000 fluorescence spectrophotometer (Hitachi, Japan).

### Stability assays

The naked VEGF siRNA (100 nM) and VEGF siRNA formed in DNWs (100 nM) was incubated in 100 µL of HeLa cell lysate solution, PBS and serum for 1 h, 2 h, 3 and 4 h, respectively. Subsequently, 15% PAGE was performed to study the stability [40].

### Cell culture

The HeLa cells (human cervix carcinoma cell), MCF-7 cells (human breast cancer cell line), HepG2 cells (human liver cancer line) and L-02 cells (human normal liver cell line) were purchased from Shanghai Institutes for Biological Sciences (SIBS) (Shanghai, China) and cultured in DMEM supplemented with 10% FBS, streptomycin (100 µg/mL) and penicillin-streptomycin (100 µg/mL) at 37 °C under 5% CO_2_ humid atmosphere. The cells were counted using a cell counting chamber (ThermoFisher scientific, USA) before each assay.

### Confocal laser scanning microscopy imaging

The HeLa cells, HepG2 cells, MCF-7 cells and L-02 cells were seeded in 48-well plate (3 × 10^4^ cells/well, 500 µL) and cultured in DMEM for 12 h, respectively. According to the manufacturer instructions, HeLa cells were transfected with DNA circuit and R-circuit, respectively, in which the DNA circuit contains H1, H2/PS and H3 to generate VEGF siRNA triggered by miR-21, and R-circuit contains random DNA (H1-R, H2-R and H3-R) that is not responsive to miR-21. In addition, the antisense miR-21 pretreated HeLa cells were transfected with DNA circuit. The final concentration of each DNA hairpin was 200 nM. After incubation for 4 h at 37 °C, the cells were washed with PBS for three times, and the fluorescence images were recorded on a Nikon Confocal Microscope A1 (Nikon, Japan) (λ_ex_ = 488 nm and λ_em_ = 520 ± 20 nm). For nuclear localization imaging, the HeLa cells were incubated with DNA circuit (200 nM for each DNA hairpin) for 4 h, followed by staining with Hoechst 33342 solution (5 µg/mL) for 30 min. After washing with 1× PBS for three times, the fluorescence images were recorded with an excitation wavelength of 405 nm and an emission wavelength of (475 ± 25) nm using a Nikon Confocal Microscope A1.

### Flow cytometry

The HeLa cells, HepG2 cells, MCF-7 cells and L-02 cells were seeded in 6-well plates (5 × 10^5^ cells /well) for 12 h and then transfected with DNA circuit for 4 h, respectively. Then, the cells were washed three times with 1× PBS and detached from the plate by trypsin. Finally, the flow cytometry assays were performed on a CytoFLEX system (Beckman Coulter, US) under the excitation of 488 nm.

### Quantitative real-time PCR

The HeLa cells and HepG2 cells were seeded in 6-well plates (5 × 10^5^ cells /well) for 12 h and then transfected with different samples (PBS, naked siRNA, DNA circuit, C-circuit, R-circuit and Lipo) for 48 h at 37 °C, respectively. C-circuit contains H1-C, H2-C and H3-C to generate negative control siRNA triggered by miR-21. The final concentration of each DNA hairpin was 200 nM. The total RNAs were extracted from the transfected cells using Trizol reagent (Invitrogen, USA). Then, the cDNA was generated using PrimeScriptRT reagent kit (Takara, Japan). Finally, the qRT-PCR analysis was performed using TB Green Premix Ex Taq™ II (TaKaRa, Japan) according to the manufacturer instructions. The PCR primer sequences are listed in Additional file 1: Table S1. The operation conditions of PCR were as follows: an initial step (95 °C for 30 s), followed by 40 cycles (95 °C for 5 s and 60 °C for 30 s). The data were analyzed by normalizing to the expression of GAPDH and using the 2^−ΔΔCT^ method. The process of RNA extraction was performed on ice.

### Western blot assay

The protein expression of cells was examined using western blot. In brief, the HeLa cells and HepG2 cells were seeded in 6-well plates (5 × 10^5^ cells/well) for 12 h and then transfected with different samples (PBS, naked siRNA, DNA circuit, C-circuit, R-circuit and Lipo) for 48 h at 37 °C. The final concentration of each DNA hairpin was 200 nM. Then, the cells were lysed in RIPA (Radio Immunoprecipitation Assay) lysis buffer for 30 min on ice and then scraped immediately. The extracted solution was transferred to an EP tube and centrifuged at 4 °C for 15 min (12,000 r/min). The concentration of the total protein was measured using a BCA protein assay kit (CoWin Biosciences, Beijing, China). A 25 µg total protein of each sample were loaded on 10% sodium dodecyl sulfate-polyacrylamide gel electrophoresis (SDS-PAGE) and electro-transferred to polyvinylidene fluoride (PVDF) membrane. After being blocked with PBS buffer containing 5% nonfat dry milk for 2 h, the membranes were incubated with the rabbit anti-VEGFA polyclonal antibody (1:1000 dilution) (Absin Bioscience Inc, Shanghai, China) and β-actin (Santa Cruz Biotechnology, USA) overnight at 4 °C, followed by incubation with goat anti-rabbit IgG-HRP secondary antibody (Absin Bioscience Inc, Shanghai, China) (1:5000 dilution) for another 1 h at room temperature. The protein bands were visualized with a Sparkjade ECL super (Sparkjade Biotechnology, Jinan, China) using Vilber Fusion FX7 Spectra (France).

### Cell apoptosis experiments

The HeLa cells and HepG2 cells were seeded in 6-well plates (5 × 10^5^ cells/well) for 12 h and then transfected with different samples (PBS, naked siRNA, DNA circuit, C-circuit, R-circuit, and Lipo) for 48 h at 37 °C. The final concentration of each DNA hairpin was 200 nM. After washing three times with 1× PBS, the collected cells were stained using Annexin V-FITC/PI Apoptosis Detection Kit according to the manufacturer instructions. Finally, the fluorescence signals of cells were analyzed using Cytomics FC 500 (Beckman, USA).

### CCK-8 assay

The HeLa Cells and HepG2 cells were seeded in 96-well plates (5 × 10^3^ cells/well) for 12 h and then transfected with different concentrations of DNA hairpins (50 nM, 100 nM, 150 nM and 200 nM) for 48 h, respectively. After washing twice with PBS, 100 µL of 10% CCK-8 solution (10 µL of CCK-8 reagent and 90 µL of DMEM without FBS) was added into each well and incubated with cells for 30 min. Then, the optical density (OD) at 450 nm was measured using a microplate reader (TECAN Safire 2, Switzerland). The relative cell viability (%) was calculated by (A_test_ − A_blank_)/(A_control_ − A_blank_) × 100%, in which A_test_, A_blank_ and A_control_ represent the OD_450_ of experimental group, blank group and control group, respectively. In addition, to demonstrate the therapeutic efficiency of in situ generated VEGF siRNA, the HeLa cells and HepG2 cells were transfected with different samples (PBS, naked siRNA, DNA circuit, C-circuit, R-circuit and Lipo), followed by determining the relative cell viability using the above methods.

### Hemolysis experiment

To assess the biocompatibility of the proposed DNA circuit in circulation, the hemolysis experiment has been performed according to the reported literature [47]. Briefly, 0.75 mL of blood was obtained from the BALB/c nude mice. The red blood cells (RBCs) were collected via centrifuging at 1000 rpm for 5 min, followed by suspended in PBS with a concentration of 5% (v/v). Then, 0.25 mL of DNA circuit was added into 0.25 mL of the diluted RBCs. The final concentrations of DNA circuit were 50 nM, 100 nM, 150 nM and 200 nM, respectively. Meanwhile, the same amount of RBCs incubated with PBS was served as the negative control, while H_2_O was acted as the positive control. After incubated at 37 °C for 4 h, the samples were centrifuged at 1000 rpm for 5 min and the supernatant were collected. Subsequently, the absorbance of supernatant at 540 nm was measured using the multimode microplate reader (Bio Tek Inc., USA). The percentage of hemolysis was calculated by the equation of Hemolysis (%) = (A_Sample_ − A_Negative control_)/(A_Positive control_ − A_Negative control_) × 100%, in which A_Sample_, A_Negative control_ and A_Positive control_ present the absorbances of RBC solution treated with DNA circuit, PBS and H_2_O, respectively.

### In vivo antitumor efficacy

All animal experiments were carried out in agreement with the Institutional Animal Care and Use Committee. The female BALB/c nude mice (5–6 weeks) were acquired from Beijing Vital River Laboratory Animal Technology Co., Ltd (Beijing, China) and randomly divided into five groups with four mice per group. The tumor xenograft models were developed by subcutaneous inoculation with 6 × 10^6^ HeLa cells suspended in 100 µL of PBS. When the tumor volumes grew to 75 mm^3^, the groups were intratumorally injected with 50 µL of PBS, naked siRNA, DNA circuit, C-circuit and R-circuit every 2 days for eight treatments, respectively (the equivalent siRNA dose for each mouse is 0.25 mg/kg). Meanwhile, the bodyweight and tumor growth were monitored during therapy. The tumor volumes were calculated using the formula of V = (L × W^2^)/2, where L and W represent the length and width of tumor, respectively. At the end of the experiment, the mice were sacrificed. In addition, the pathological changes of tumors were analyzed using hematoxylin and eosin (H&E) staining assay and terminal deoxynucleotidyl transferase-mediated dUTP nick-end labeling (TUNEL) staining. To further study the biocompatibility of the proposed DNA circuit in vivo, the H&E assay was performed to analyze the pathological changes of major organs including heart, liver, spleen, kidney and lung from mice in different groups. The images were obtained with Pannoramic MIDI (3D HISTECH Ltd., Hungary).

### Statistical analysis

The data was expressed as mean ± standard deviations. We used one-way analysis of variance (ANOVA) to analyze the statistical difference (* means *P* ˂ 0.05, ** means *P* ˂ 0.01, and *** means *P* ˂ 0.001).

## Results and discussion

### Principle of miRNA activated enzyme-free DNA circuit

**Scheme 1:**
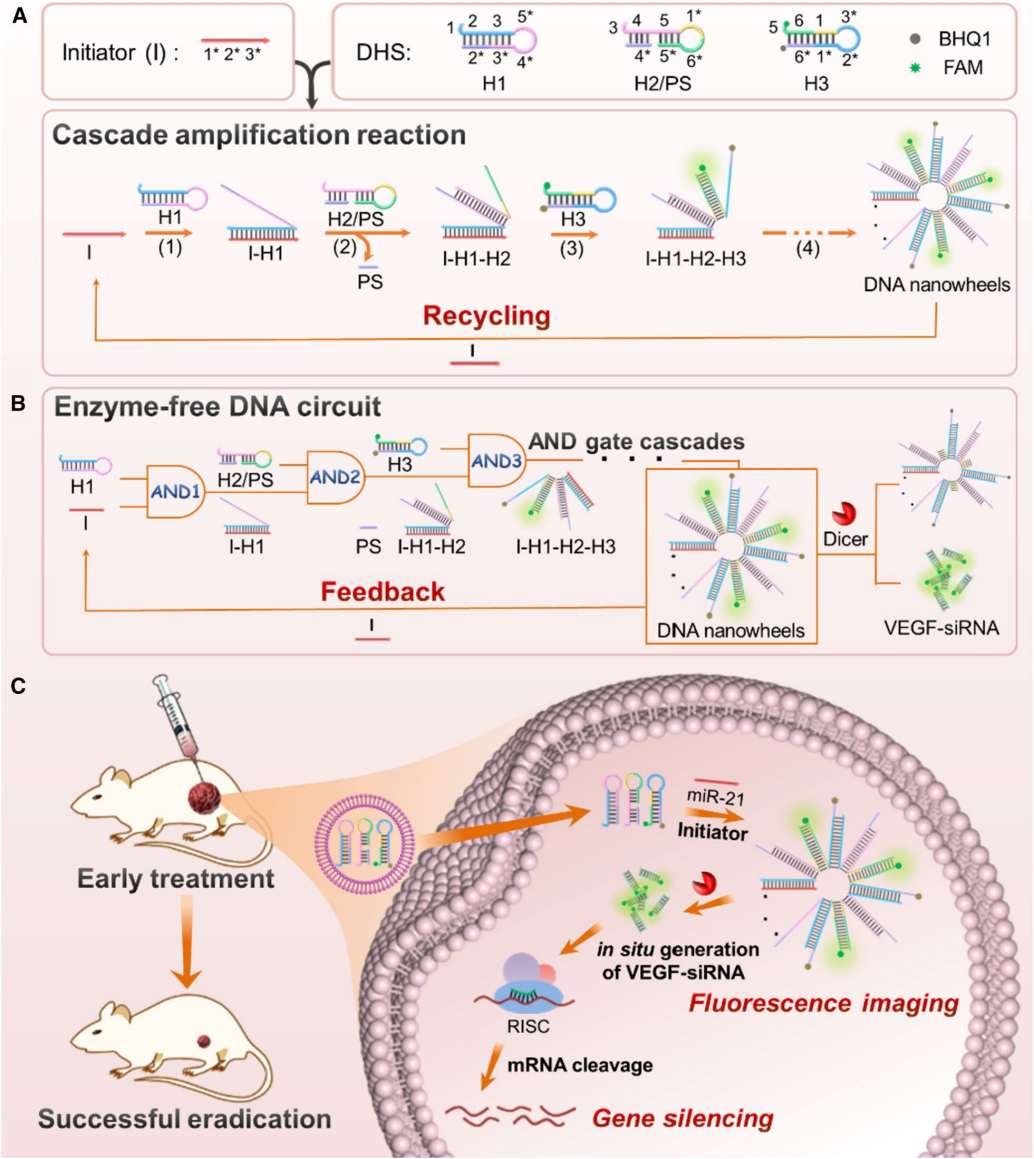
Schematics of the logic reaction pathways and application of the enzyme-free DNA circuit for visualization of miR-21 in living cells and in situ generation of siRNA for enhanced gene therapy of cancers

The principle of miRNA activated enzyme-free DNA circuit for in situ generation of siRNA for gene therapy is illustrated in Scheme 1. In this study, miR-21 which is closely related to the onset, progression, and prognosis of many cancers is chosen as the model target. As shown in Scheme 1A, three kinds of DNA hairpins (H1, H2 and H3) and a PS are rationally designed, in which H1 is served as the recognition probe for miR-21, while VEGF antisense and sense sequences are incorporated into H2 and H3, respectively. To protect the antisense sequence of VEGF-siRNA, PS hybridizes with H2 to form the H2/PS hybrid. To facilitate FRET, fluorophore (6-Carboxyfluorescein, FAM) and quencher (Black Hole Quencher-1, BHQ1) are labeled at 3′ and 5′ ends of H3, respectively. In the absence of miR-21, H1, H2/PS and H3 can metastably coexist, and no fluorescence signal is observed due to the proximity of FAM and BHQ1 in H3. Upon the introduction of miR-21, it simultaneously serves as the initiator (I) and catalyst to activate the operation of DNA circuit via catalytic hairpin assembly based on the strand displacement reaction, resulting in the formation of “DNWs” along with the recovery of fluorescence signal. In brief, miR-21 first binds to the toehold domain 1 of H1 and undergoes a DNA toehold-mediated strand displacement reaction to open H1 (Scheme 1A, reaction 1). Then, the newly exposed single-stranded region of H1 hybridizes with domain 3 of H2/PS, replacing PS strands to form the I/H1/H2 hybrid (Scheme 1A, reaction 2). Subsequently, the toehold domain 5* of H3 hybridizes with I/H1/H2 hybrid, opening the hairpin structure of H3 (Scheme 1A, reaction 3). Analogously, the unfolded region of H3 (1*–2*–3*) again opens H1 to trigger the cascaded self-assembly reactions among DNA hairpins until miR-21 is displaced, leading to the formation of “DNWs” (Scheme 1A, reaction 4). Meanwhile, the released miR-21 is reusable in another reaction cycle to make the self-assembly process operate in a catalytic fashion. Based on the principle of strand displacement reaction performed in the DNA circuit, the sequences for miRNA recognition (domains 1–2–3) and siRNA generation (domains 5–6) are independently with each other. Thus, this strategy be readily applied for the response of any miRNA and in situ generation of various siRNAs only by rationally designing the DNA hairpins according to the sequences of corresponding miRNA and siRNA, which presents the universality of the proposed DNA circuit. Interestingly, the behavior of catalytic hairpin assembly is consistent with the operation of a DNA logic circuitry which consists of cascades of AND gates. Briefly, miR-21 hybridizes with H1 to activate the first AND gate (AND 1), in which miR-21 and H1 act as the inputs, while the I-H1 hybrid as the output. Then, the resulting output and H2/PS work as the inputs to trigger the operation of the second AND gate (AND 2), resulting in the generation of I/H1/H2 output. Subsequently, the third AND gate (AND 3) is activated by H3 which hybridizes with I/H1/H2 to form the hybrid I/H1/H2/H3. Since the exposed sequence of H3 is identical with miR-21, the formed output I/H1/H2/H3 can again hybridizes with H1 to initiate the following cascaded AND gates until miR-21 is replaced, resulting in the formation of “DNWs” as the output. The released miR-21 as a catalyst unit facilitate the feedback mechanism for isothermal signal amplification. In particular, the VEGF antisense and sense sequences are incorporated into H2 and H3, which allows the in situ generation of numerous VEGF siRNAs in “DNWs”. By means of endogenous enzyme (Dicer) processing, VEGF siRNA can be specifically cleaved to suppress the expression of corresponding mRNA and protein, achieving efficient gene therapy of cervical carcinoma (Scheme 1B).

### Feasibility of enzyme-free DNA circuit

**Fig. 1:**
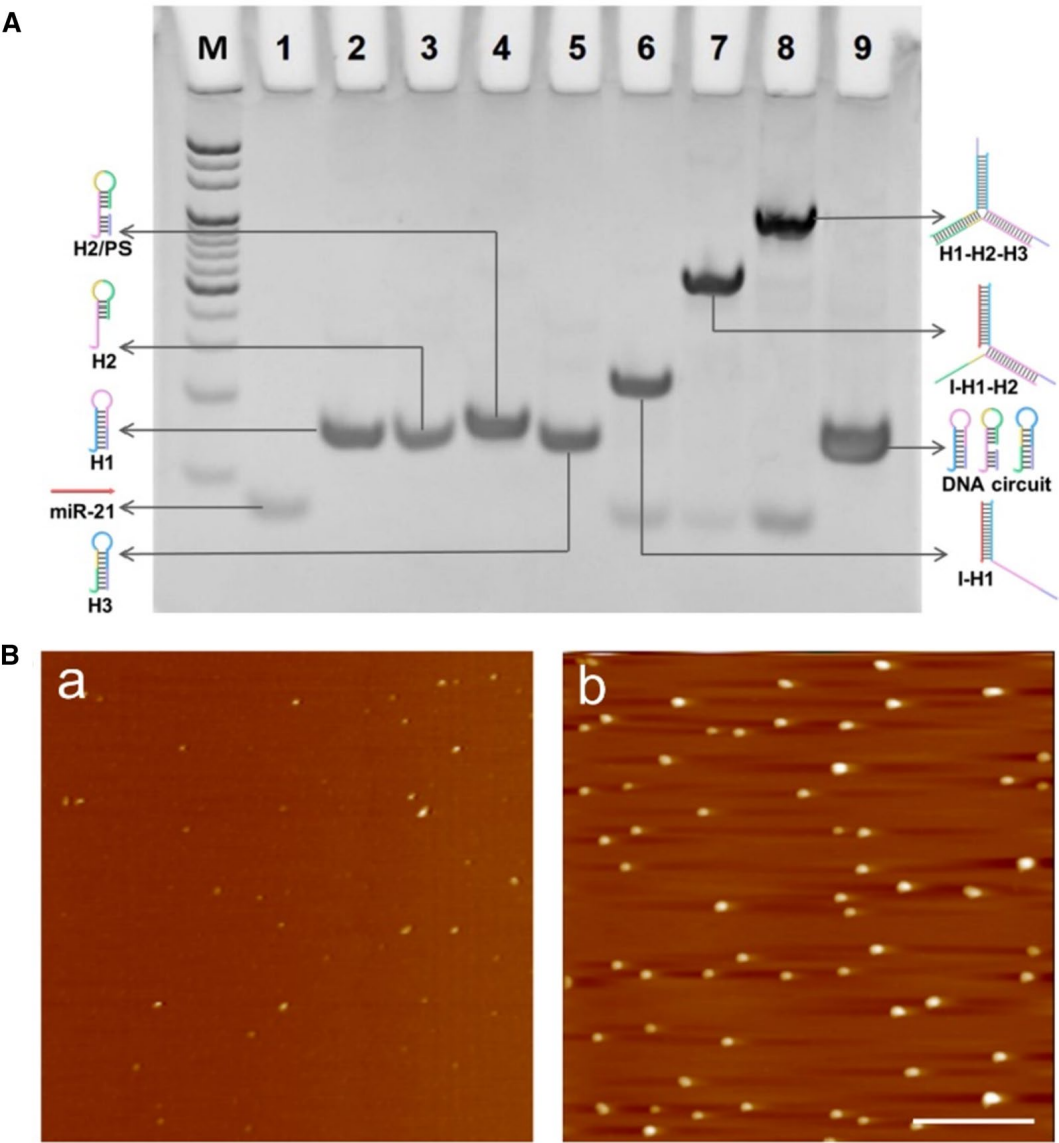
Characterization of the enzyme-free DNA circuit triggered by miR-21. **A** Native PAGE. Lane M: 20 bp DNA maker; Lane 1: miR-21; Lane 2: H1; Lane 3: H2; Lane 4: H2/PS; Lane 5: H3; Lane 6: miR-21 + H1; Lane 7: miR-21 + H1 + H2/PS; Lane 8: miR-21 + H1 + H2/PS + H3; Lane 9: H1 + H2/PS + H3 (control). The initial concentration of each hairpin is 10 µM. **B** AFM images of DNA circuit (H1 + H2/PS + H3) (a) without and (b) with miR-21, respectively. Scale bar: 100 nm

The reaction pathways of the proposed enzyme-free DNA circuit triggered by miR-21 are first verified using PAGE (Fig. 1A). The slow mobility of H2/PS hybrid (lane 4) confirms its successful formation compared with H2 (lane 3). When H1 is mixed with the equal amount of miR-21, a new band corresponding to I/H1 is generated because miR-21 opens the hairpin structure of H1 through toehold-mediated strand displacement reaction (lane 6). Upon the introduction of H2/PS, a new band represented the I/H1/H2 hybrid is produced (lane 7). After addition of H3 into I/H1/H2, the reaction products show two bands with much low and high mobility, respectively, demonstrating the generation of DNA assembly along with the release of miR-21 for recycling (lane 8). In contrast, in the absence of miR-21, no new band is observed, indicating the excellent specificity of the enzyme-free DNA circuit in response to miR-21 and there is no leakage without target (lane 9). The feasibility of this miRNA-responsive DNA circuit was further characterized by AFM (Fig. 1B). In the absence of miR-21, only some small spots are observed, indicating no assembled product is yielded. In contrast, a large amount of monodispersed spherical-like structures with an average height of 18 nm are appeared upon the introduction of 0.1× miR-21 into the system. This phenomenon is well agreed with the results of native PAGE, which further confirm the performance of DNA circuit as expected. In addition, we have used the fluorescence assay to verify the operation of DNA circuit consisted of cascaded AND logic gates. There are four inputs (I, H1, H2/PS and H3) in this DNA circuit, while H3 is labeled with fluorophore/quencher pairs (FAM/BHQ1) to generate output signal. The inputs are defined as 1 when they are present, otherwise as 0 when they are absence; and the output is defined as 1 when the fluorescence intensity exceeds a threshold value of 200, otherwise as 0. The experimental results and the corresponding truth table are shown in Additional file 1: Fig. S1. Only when all the four inputs (I, H1, H2/PS and H3) are present, the DNA circuit can be triggered for the assembly of DNWs, resulting in the recovery of fluorescence signal of FAM. In contrast, no obvious fluorescence signals are recorded in the control groups containing other input combinations, which is consistent with the performance of cascaded AND gate.

### In vitro detection of miR-21

**Fig. 2:**
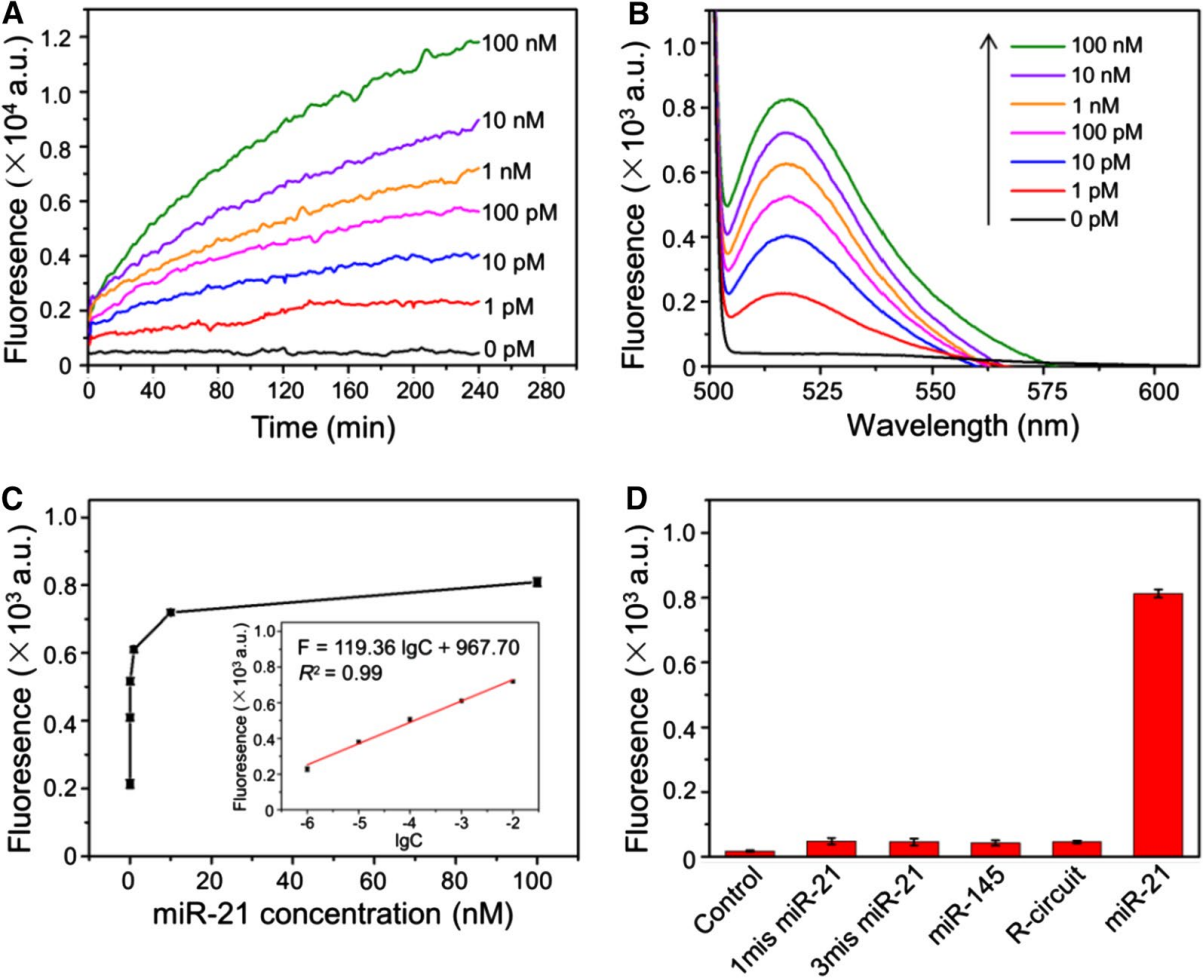
Sensitivity and selectivity of the enzyme-free DNA circuit triggered by miR-21. **A** Fluorescence kinetics of the DNA circuit triggered by various concentrations of miR-21 (from 1 pM to 100 nM). **B** The corresponding fluorescence spectra at 4 h. **C** Relationship of the fluorescence intensity at 520 nm to miR-21 concentration. Inset: the linear fit between the fluorescence intensity at 520 nm and the logarithmic value of miR-21 concentration. Error bars represent the standard deviation of three experiments. **D** Specificity of the DNA circuit in response to different analytes (final concentration is 100 nM)

First, the kinetics of the proposed DNA circuit was studied by labeling fluorophore (FAM) and quencher (BHQ1) at 3′ and 5′ ends of H3, respectively. Upon the introduction of miR-21 with different concentrations, the real-time fluorescence is monitored with a 30 s interval for 4 h (Fig. 2A). In the absence of miR-21, the fluorescence of FAM is quenched by BHQ1. In contrast, the fluorescence intensity gradually increases with increasing the concentration of miR-21, which demonstrates the high target dependence of DNA circuit. Then, miR-21 was quantitatively detected by measuring the fluorescence spectra after treated with different concentrations of miR-21 for 4 h. As shown in Fig. 2B, the fluorescence intensities are gradually enhanced with the increase of miR-21 ranging from 1 pM to 100 nM. A good linear relationship is presented between miR-21 concentration and fluorescence intensity at 520 nm with a detection limit of 0.25 pM (3σ) (Fig. 2C).

Further, to verify the specificity of the proposed enzyme-free DNA circuit, different targets, including single-base mismatched miR-21 (1mis miR-21), three-base mismatched miR-21 (3mis miR-21), miR-145 and miR-21 are analyzed, respectively (Fig. 2D). As expected, in comparison to the control group without any target, only miR-21 induces an intensified fluorescence. When the recognition domain in H1 is replaced with the non-specific sequence for miR-21 (the sequences of H2 and H3 are changed accordingly) (R-circuit), no enhanced fluorescence is observed, which further proves that the operation of this DNA circuit is highly dependent on the specific Watson–Crick base pairing. Given the high sensitivity and specificity, our proposed enzyme-free DNA circuit in the response to miR-21 has a promising prospect for further application in complex cellular environment.

### MiR-21 imaging in living cells

**Fig. 3:**
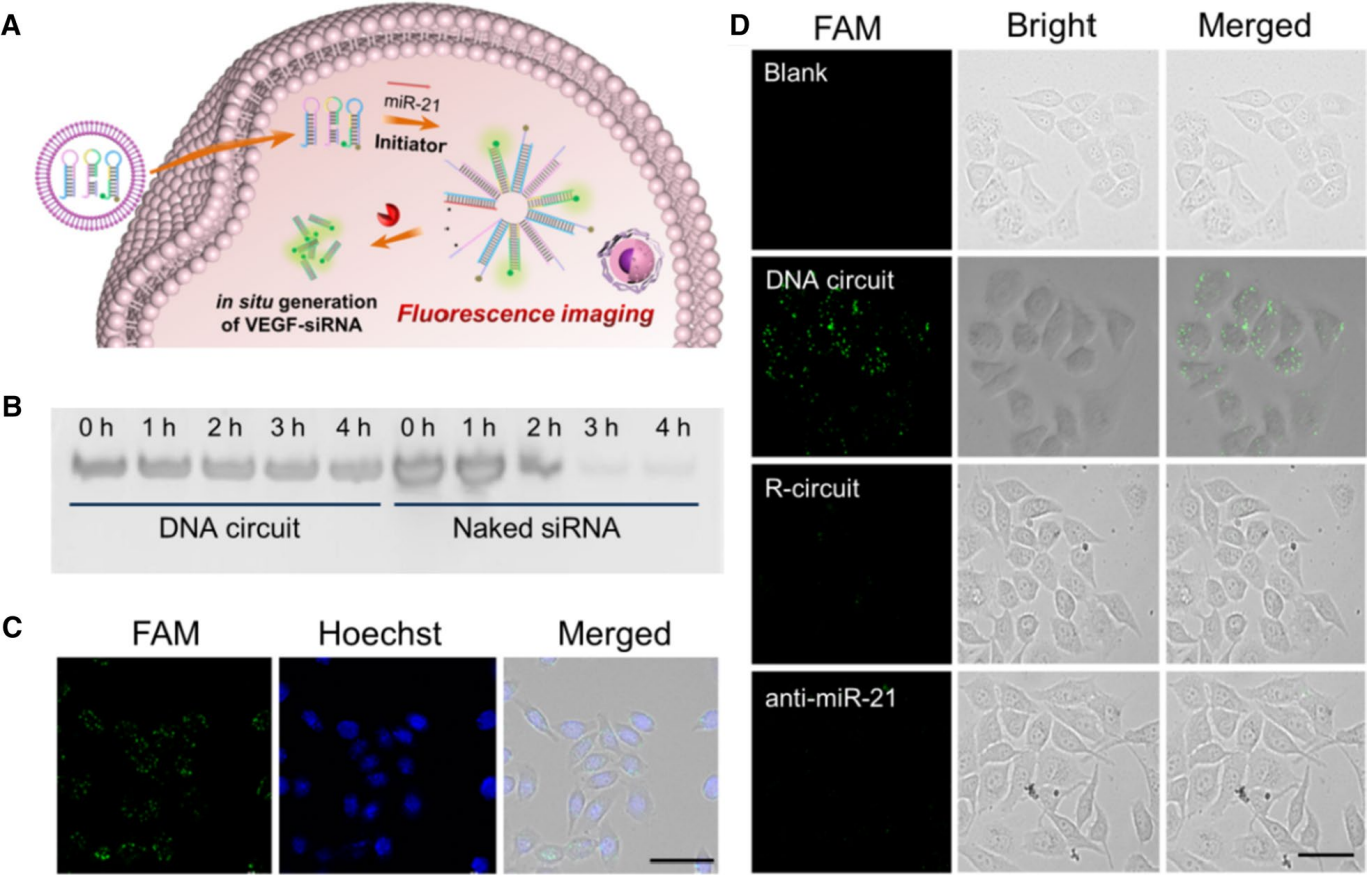
Visualization analysis of intracellular miR-21. **A** Schematics of in situ imaging of miR-21 in living cells. **B** Stability of DNA circuit and naked siRNA after incubation with cell lysate for 1 h, 2 h, 3 and 4 h, respectively. **C** Nuclear localization images of HeLa cells after incubated with DNA circuit for 4 h. **D** CLSM images of HeLa cells with different treatments. The final concentration of each DNA hairpin is 200 nM. Scale bars in **C** and **D**: 50 μm

In this work, the DNA circuit is transfected into living cells by liposome, in which the loading content and efficiency are calculated using UV-vis spectrophotometry (Additional file 1: Fig. S2). It has been reported that miR-21 is overexpressed in Hela cells [48]. Thus, Hela cells were chosen as the model to investigate the intracellular operation of this enzyme-free DNA circuit (Fig. 3A). We first evaluated the biostability of DNA circuit using PAGE, which is a fundamental feature of biomaterials for in vivo applications. As shown in Fig. 3B, after the DNA circuit is triggered by miR-21 with a ratio of 1:1, nearly no degradation is observed when the products are incubated with cell lysate even for 4 h. In contrast, the naked siRNAs are gradually degraded with prolong of time. Moreover, the DNA circuit which are incubated with human serum and PBS buffer with (pH 7.4) are further characterized by PAGE (Additional file 1: Fig. S3). All the results suggest that the DNA hairpins can well protect the siRNA sequences from degradation in complex physiological environment. Then, the colocalization experiments were carried out by staining cell nucleus with Hoechst 33342 to locate the distribution of miR-21 in HeLa cells. The green fluorescence of FAM can be readily observed at the nuclear periphery, illustrating the distribution of miR-21 in the cytoplasm which initiates the DNA circuit and results in the recovered fluorescence (Fig. 3C). To further demonstrate the feasibility of the proposed method in living cells, the HeLa cells were transfected with DNA circuit and R-circuit, respectively. After incubated for 4 h, the fluorescence can be easily observed in DNA circuit treated cells. However, since the intracellular miR-21 cannot initiate the assembly among hairpins with R-circuit, almost no fluorescence is generated. In addition, the HeLa cells pretreated with antisense miR-21 also show an extremely weak fluorescence after transfected with DNA circuit (Fig. 3D). These results indicate the successful operation of this enzyme-free DNA circuit in living cells, and the fluorescence signals are indeed produced triggered by miR-21.

To demonstrate the expression level of miR-21 in different cells, HeLa cells (human cervix carcinoma cell line), HepG2 cells (human liver cancer cell line), MCF-7 cells (human breast adenocarcinoma cell line) and L-02 cells (human normal liver cell line) are treated with the DNA circuit, respectively. It has been reported that the amount of miR-21 in HeLa, MCF-7 and HepG2 cells is higher than that in L-02 cells [49]. Accordingly, an obvious fluorescence recovery is observed in HeLa, MCF-7 and HepG2 cells, while nearly no fluorescence is appeared in L-02 cells (Additional file 1: Fig. S4a). Notably, a stronger fluorescence signal is presented in MCF-7 cells than that of HeLa cells, which can be mainly contributed to the higher endogenous expression level of miR-21 in MCF-7 cells. This result is in agreement with previous work [50]. Also, flow cytometry assay was carried out to detect the fluorescence changes (Additional file 1: Fig. S4b). Compared with L-02 cells, the HeLa cells, MCF-7 cells and HepG2 cells treated with DNA circuit demonstrate the significantly enhanced fluorescence signals. which further confirm the practicability and reliability of the proposed DNA self-assembly strategy for in situ imaging of miRNA in living cells.

### Gene silencing and in vitro anticancer efficacy

**Fig. 4:**
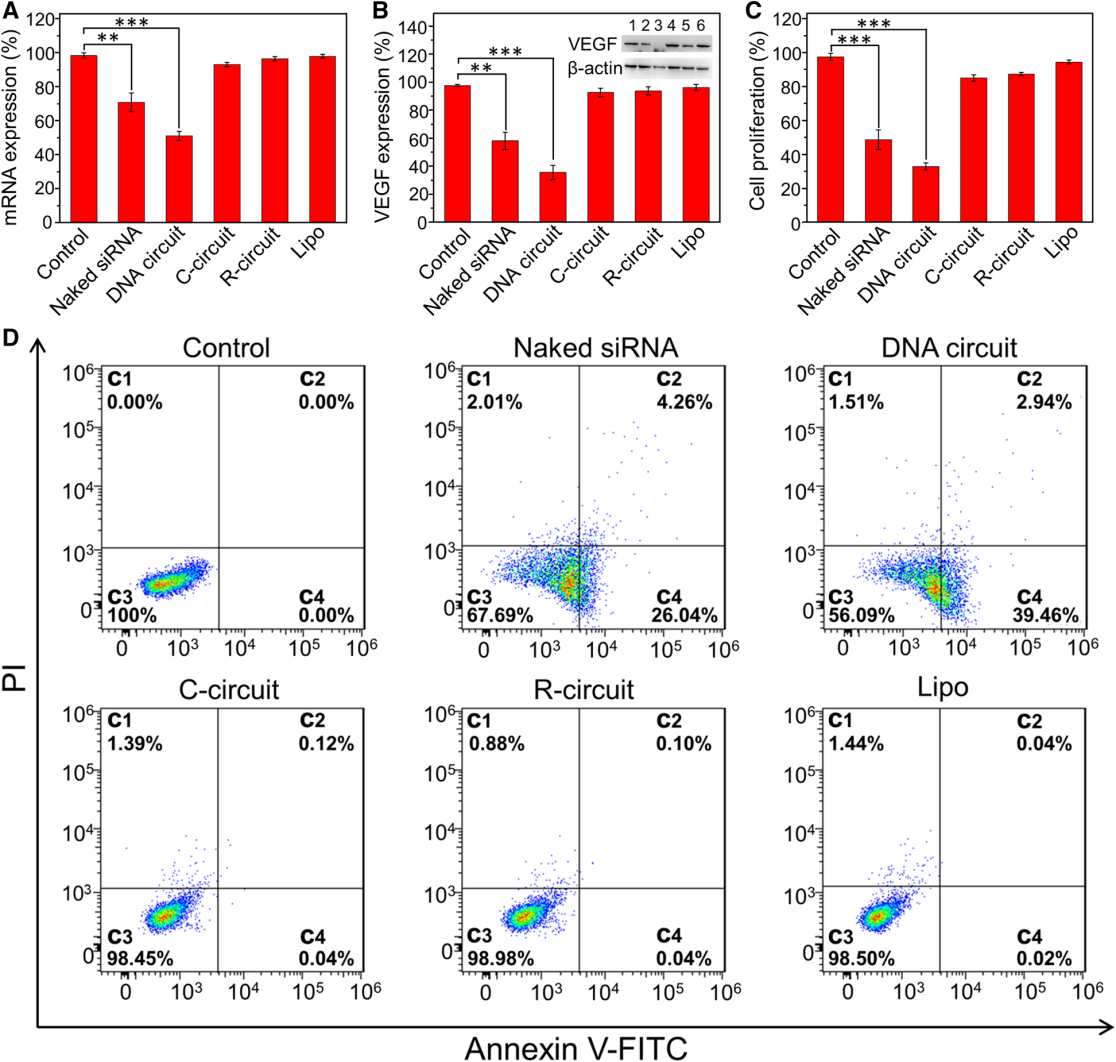
Intracellular therapeutic effect of the DNA circuit on HeLa cells. **A** VEGF mRNA expression, **B** VEGF protein expression, **C** proliferation and **D** apoptosis assays of HeLa cells after different treatments. Inset in **B** shows the western blot images. Lane 1: control; Lane 2: naked siRNA; Lane 3: DNA circuit; Lane 4: C-circuit; Lane 5: R-circuit; Lane 6: Lipo. The HeLa cells without any treatment are the control. The final concentration of each DNA hairpin is 200 nM. The data error bars indicate means ± SD (n = 3). **P < 0.01, ***P < 0.001 (two-tailed Student’s t test)

The studies have reported that VEGF is a critical regulator of tumor-induced angiogenesis, which facilitates tumor growth and survival [51, 52]. Thus, we chose VEGF as the target gene in this work to evaluate the gene silencing and anticancer efficacy via in situ generation of VEGF siRNA. To investigate the gene silencing efficacy, the HeLa cells were incubated with naked siRNA, DNA circuit, C-circuit, R-circuit, and Lipo for 48 h, respectively. The VEGF mRNA expression was analyzed by qRT-PCR (quantitative real-time polymerase chain reaction). As shown in Fig. 4A, the HeLa cells transfected with C-circuit, R-circuit and Lipo show little inhibition effect with the VEGF mRNA expression above 93.02%. Compared with the naked siRNA-treated cells (70.78%), the VEGF mRNA expression level in HeLa cells is significantly decreased after incubated with DNA circuit (51.06%). Further, the inhibitory effect of VEGF protein expression was studied by western blot assay (Fig. 4B). As expected, the expression level of VEGF protein in DNA circuit-treated HeLa cells (35.54%) is lower than that in the naked siRNA group (58.11%). These results confirm that the in situ generation of VEGF siRNA via miR-21 triggered DNA circuit can induce an efficient gene silencing and reduce the corresponding protein expression, which provide an excellent way to improve the specificity and efficiency of gene therapy.

Subsequently, the antitumor efficacy was evaluated using cell counting kit-8 (CCK-8) assay. After incubating HeLa cells with different concentrations of DNA circuit, the cell viability reveals a concentration-dependent decrease (Additional file 1: Fig. S5). When the concentration of DNA circuit is 200 nM, the cell viability decreased to 33.09%. To further evaluate the therapeutic efficiency of in situ siRNA assembly, the HeLa cells were transfected with different samples including naked siRNA, DNA circuit, C-circuit, R-circuit and Lipo, respectively. As shown in Fig. 4C, no significant loss of viability is observed in HeLa cells after treated with C-circuit, R-circuit and Lipo, indicating the high specificity of the proposed DNA circuit. Notably, the inhibition effect of DNA circuit on Hela cells (67.14%) is obviously higher than that of naked siRNA (50.89%) due to the improved delivery stability and high reaction efficiency of DNA circuit. Moreover, the therapeutic effect is examined via cell apoptosis assays using Annexin V-FITC apoptosis detection kit (Fig. 4D). The HeLa cells treated with DNA circuit shows the highest apoptotic rate (42.40%) compared with the cells treated with naked siRNA (30.30%), C-circuit (0.16%), R-circuit (0.14%) and Lipo (0.06%). These results further demonstrate the improved stability and therapeutic effect of VEGF siRNA that are in situ generated triggered by endogenous miR-21.

Owing to the high expression of miR-21 in HepG2 cells, we further chose HepG2 cells to investigate the applicability of this enzyme-free DNA circuit for gene therapy. After transfected HepG2 cells with 200 nM DNA circuit, the expression of VEGF mRNA and the corresponding protein are inhibited to 48.04 and 41.92%, respectively (Additional file 1: Fig. S6a, b), which also verifies the excellent performance siRNAs that are in situ generated for gene silencing. In addition, a significant decrease in cell viability (53.16%) and increase in apoptosis rate (46.02%) are observed (Additional file 1: Fig. S6c, d), indicating the proposed enzyme-free DNA circuit can be used as a general treatment method for various cancer types with excellent therapeutic effect.

### In vivo tumor therapy

**Fig. 5:**
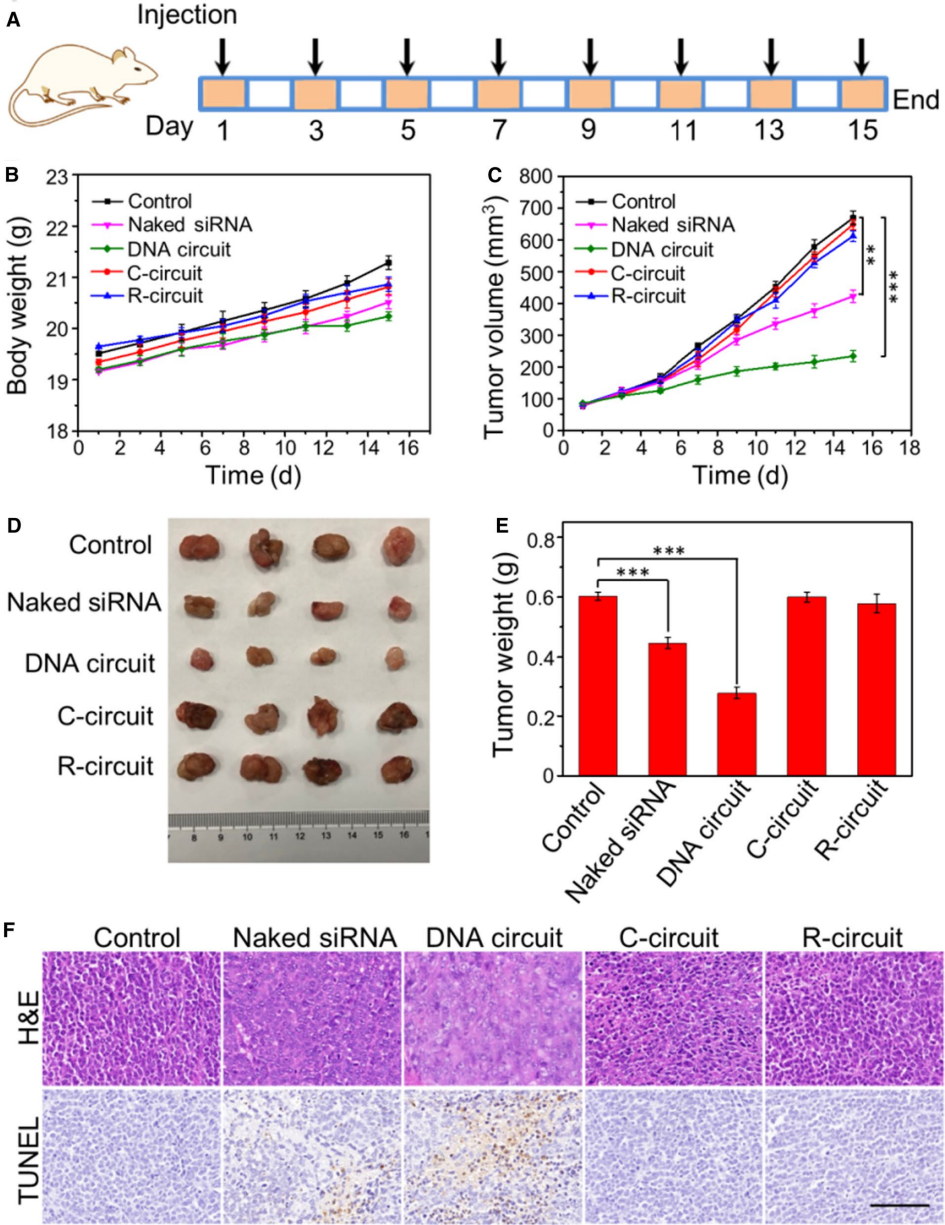
In vivo tumor therapy. **A** Time schedule for the treatment process. **B** Bodyweight and **C** tumor volume changes of mice in different groups during therapy. **D** Photographs of tumors dissected from HeLa tumor-bearing mice at the therapeutic terminal. **E** Tumor weight of mice in different groups after therapy. **F** H&E and TUNEL staining of tumor tissues with different treatments. Scale bar: 100 μm. Error bars indicate means ± SD (n = 4). **P < 0.01, ***P ˂ 0.001 (two-tailed Student’s t test)

The in vivo antitumor efficacy of DNA circuit was evaluated using HeLa xenograft tumors. Firstly, the hemolysis experiment was performed to assess the biocompatibility of the proposed DNA circuit in circulation. The experimental shows there is a negligible hemolysis in DNA circuit after treated with different concentrations, demonstrating its excellent biocompatibility (Additional file 1: Fig. S7). The mice were randomly divided into five groups (four mice per group). When the tumor volume reached ~ 75 mm^3^, the mice in each group were respectively treated with PBS, naked siRNA, DNA circuit, C-circuit and R-circuit via intratumor injection every other day for eight times, in which the dosage of siRNA was 0.25 mg/kg. In addition, the in vivo antitumor efficacy of liposome is evaluated, in which liposome shows the little inhibition on tumor growth, demonstrating its excellent biocompatibility in the indicated dosages. (Additional file 1: Fig. S8). The time schedule for treatment of tumor-bearing mice is shown in Fig. 5A. The variations of body weights in mice were monitored every 2 days d uring the treatment to evaluate the side effect of DNA circuit. From Fig. 5B, there is no obvious difference of body weight among the groups, demonstrating that the proposed DNA circuit has no significant side effect on the treated mice. During the treatment, the tumor volumes were measured to observe the tumor growth inhibition of the DNA circuit (Fig. 5C). For PBS, R-circuit and C-circuit treated groups, the tumor sizes increase rapidly, showing little inhibition on tumor growth in these cases, while the naked siRNA treated group reveals a weak inhibitory effect. Remarkably, the DNA circuit treated group exhibits the strongest antitumor effect. The photographs of mice under different treatments after 15 days are recorded (Additional files 1: Fig. S9), and the corresponding photos and the average weight of the removed tumors in different groups are shown in Fig. 5D, E, respectively. Furthermore, to test the antitumor effect of DNA circuit, the tumor tissues were analyzed with H&E staining and TUNEL assay (Fig. 5F). As expected, the tissue slices from DNA circuit group shows the most significant cell apoptosis and necrosis, demonstrating the excellent antitumor effect via in situ generation of siRNA.

**Fig. 6:**
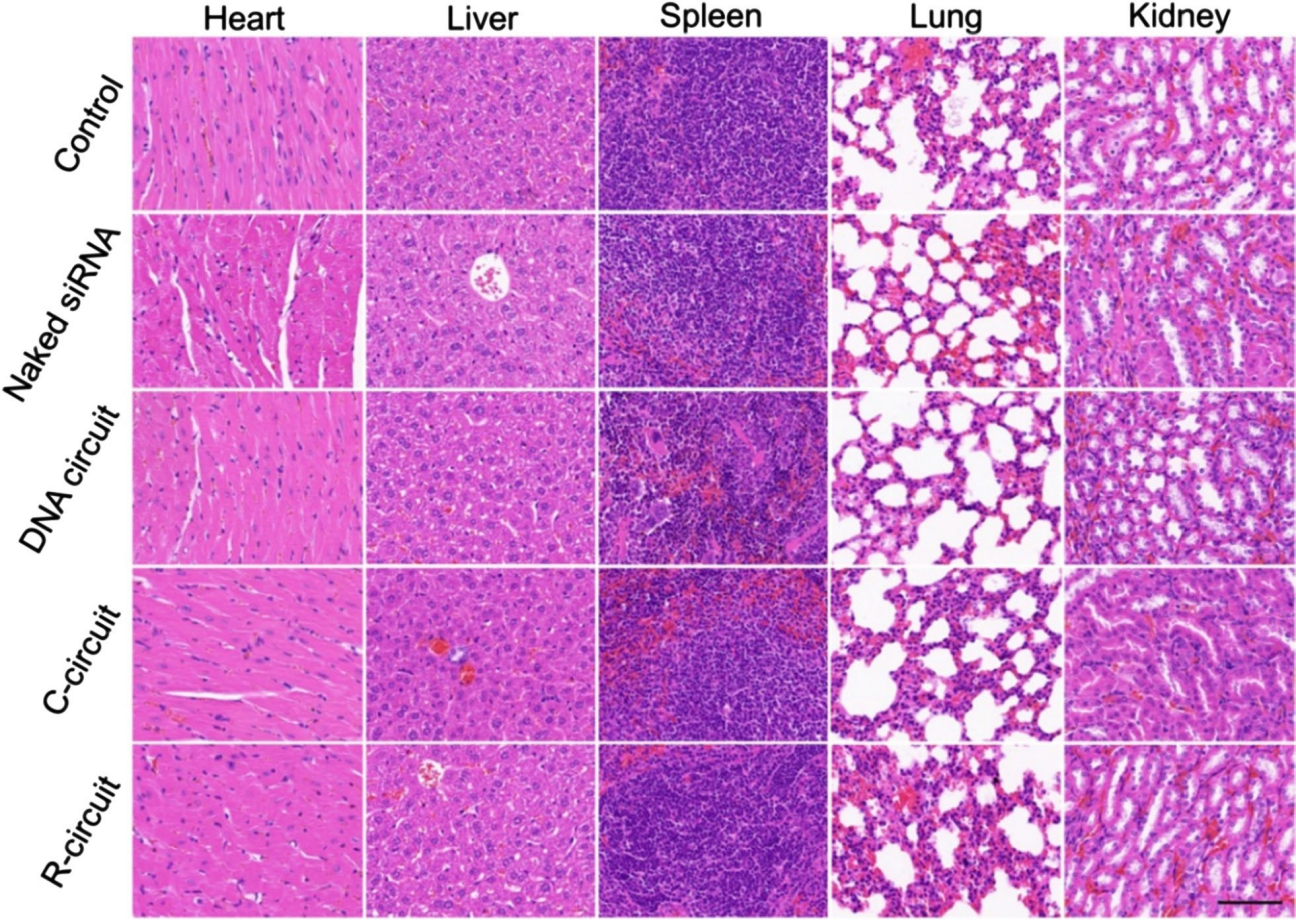
H&E staining of major organs (heart, liver, spleen, lung and kidney) from mice in different groups. Scale bar: 100 μm

Additionally, the pathological results of major organs (heart, liver, spleen, lung and kidney) were obtained by H&E staining (Fig. 6). All images exhibit the well-defined cytoplasm and nuclei, which has no noticeable abnormality compared with the PBS treated group, demonstrating the negligible side effects of DNA circuit in vivo. Therefore, the in situ generation of VEGF siRNA triggered by endogenous miR-21 provides a promising approach with the advantages of good biosafety and biocompatibility for highly efficient gene therapy of cervical carcinoma.

## Conclusions

In summary, we have proposed an endogenous miR-21-responsive enzyme-free DNA circuit for in situ generation of VEGF siRNA and efficient gene therapy of cervical carcinoma. The proposed DNA self-assembly protocol has demonstrated several unique advantages. (1) Easy operation. This strategy involves only three DNA hairpins, which can be specifically triggered by intracellular miR-21 for the formation of “DNWs” via isothermal strand displacement reaction without complicated design in sequences and the assistance of exogenous enzymes. (2) Multifunctionality. Through rational design, this work has readily realized sensitive diagnosis of cervical carcinoma upon recognized by miR-21. More importantly, the in situ generation of siRNA demonstrate better gene therapy efficiency than that of naked siRNA due to the increased stability of siRNA protected by DNA hairpin structures and the high reaction efficiency of DNA circuit. (3) Universality. Based on the principle of strand displacement reaction performed in the DNA circuit, the sequences for miRNA recognition and siRNA generation are independently with each other. Thus, this strategy be readily applied for the response of any miRNA and in situ generation of various siRNAs only by rationally designing the DNA hairpins according to the sequences of corresponding miRNA and siRNA. Overall, our proposed enzyme-free DNA circuit provides a new insight into the construction of multifunctional DNA nanomaterials, which holds great potential in nanotheranostics as well as precision and personalized medicine.

## Supplementary Information

**Additional file 1.** Additional figures and tables.

## Data Availability

All data and materials of this study can be obtained from the corresponding author upon request.

## Abbreviations

DNWs: DNA nanowheels
FRET: Fluorescence resonance energy transfer
FAM: 6-Carboxyfluorescein, FAM
BHQ: Black Hole Quencher-1
VEGF: Vascular endothelial growth factor
PCR: Polymerase chain reaction
RCA: R olling circle amplification
FBS: Fetal bovine serum
DMEM: Dulbecco’s modified Eagle’s Medium
TEMED: *N*,*N*,*N*′,*N′*-Tetramethyl ethylenediamine
PS: protect strand
AFM: Atomic Force Microscopy
SIBS: Shanghai Institutes for Biological Sciences
CLSM: Confocal laser scanning microscopy
SDS-PAGE: Sodium dodecyl sulfate-polyacrylamide gel electrophoresis
PVDF: Electro-transferred to polyvinylidene fluoride
H&E: Hematoxylin and eosin
TUNEL: Terminal deoxynucleotidyl transferase-mediated dUTP nick-end labeling
HeLa cells: Human cervix carcinoma cell line
HepG2 cells: Human liver cancer cell line
L-02 cells: Human normal liver cell line
qRT-PCR: Quantitative real-time polymerase chain reaction
CCK-8: Cell counting kit-8
