# Supplementary material for: Endogenous microRNA triggered enzyme-free DNA logic self-assembly for amplified bioimaging and enhanced gene therapy via in situ generation of siRNAs
Source: J Nanobiotechnology. 2021 Sep 26;19:288. doi: 10.1186/s12951-021-01040-x (PMC8474761; doi:10.1186/s12951-021-01040-x)
Supplement: Supplementary file 1 — Additional file 1. Additional figures and tables. [file 12951_2021_1040_MOESM1_ESM.docx]

**Additional file 1**

**Endogenous microRNA triggered enzyme-free DNA logic self-assembly for amplified bioimaging and enhanced gene therapy via in situ generation of siRNAs**

Qinghua Jiang^1,#^, Shuzhen Yue^2,#^, Kaixin Yu^2^, Tian Tian^1^, Jian Zhang^2^, Huijun Chu^1^, Zhumei Cui^1,^*, Sai Bi^1,2^*

^1^Department of Obstetrics and Gynecology, The Affiliated Hospital of Qingdao University, Qingdao, 266003, PR China

^2^College of Chemistry and Chemical Engineering, Qingdao University, Qingdao, 266071, PR China

*Corresponding authors. E-mail: cuizhumei1966@126.com; [bisai11@126.com](mailto:bisai11@126.com)

^#^These authors contribute equally to this work.


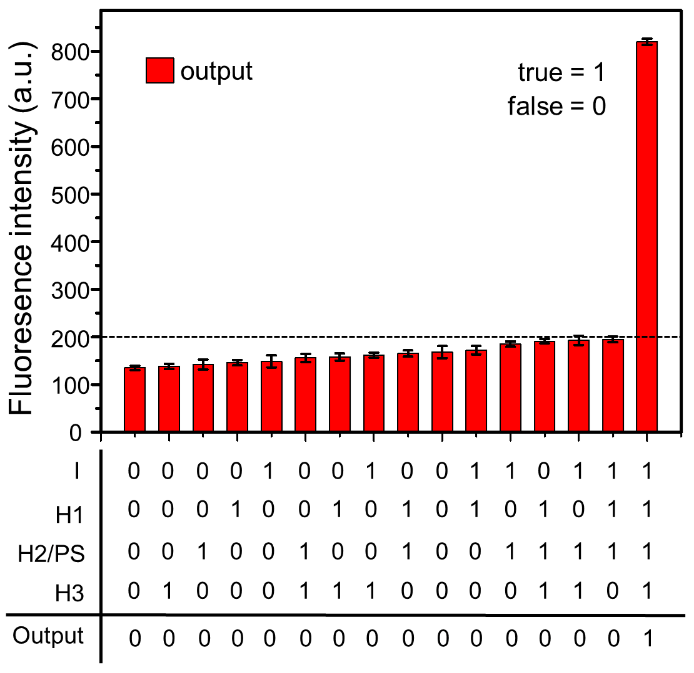


**Fig. S1.** Fluorescence intensities and truth table of the DNA circuit consisted of cascaded AND logic gates. The concentration of each kind of DNA is 10 µM. The threshold fluorescence value is indicated by the red line.

**Calculation of loading content and efficiency of liposome**

First, the DNA circuits with different concentrations (from 0 nM to 300 nM) are prepared using TE buffer, and the corresponding UV-vis absorbance at 260 nm is recorded. The regression equation is shown as A = 1.01 × 10^-3^C + 1.04 ×10^-4^, in which C and A represent the concentration of DNA circuit and the corresponding UV-vis absorbance, respectively (n = 3, *R*^2^ = 0.99) (Fig. S2).


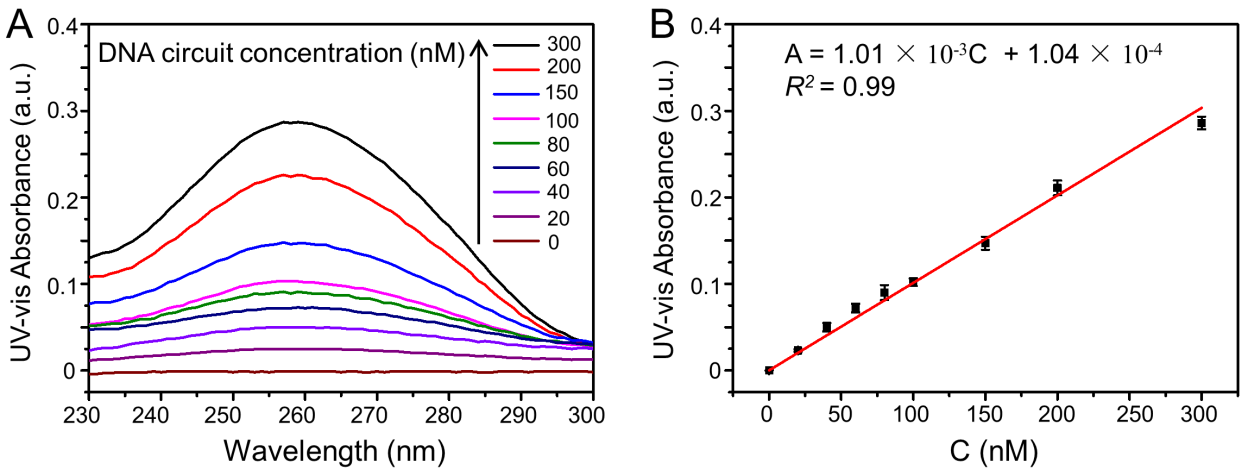


**Fig. S2.** (A) UV-vis absorbance spectrum and (B) corresponding standard curve of DNA circuit with different concentrations. Error bars represent the standard deviation of three experiments.

Then, the DNA circuit solution (200 nM) is incubated with the liposome (4 µL) for 15 min, followed by centrifugation at 10,000 rpm for 10 min. Subsequently, the supernatant is collected. The loading content and efficiency of DNA circuit can be quantitatively calculated according to the recorded UV-vis absorbance of DNA solution at 260 nm before and after incubation.

UV-vis absorbance of unloaded DNA circuit in the supernatant: A_1_ = 0.063

The corresponding concentration: C_1_ = 62.27 nM.

Volume of DNA solution: V = 200 µL

Moles of total DNA circuit loaded into liposome: n_loaded DNA circuit_ = (C_0_ - C_1_) × V= (200 – 62.27) × 10^-9^ × 2.0 × 10^-4^ = 2.75 × 10^-11^ moles

Total moles of DNA circuit: n_total DNA circuit_ = C_0_ × V= 4.00 × 10^-11^ moles

The loading efficiency of DNA circuit = n_loaded DNA circuit_ /n_total DNA circuit_ × 100% = 2.75 × 10^-11^ /4.00 × 10^-11^ × 100% = 68.8%.

Thus, the loading content and efficiency are 2.75 × 10^-11^ moles and 68.8%, respectively.

**Stability of DNA circuit in PBS and serum**

To investigate the stability of DNA nanomaterials, the DNA circuit and naked siRNAs are incubated with PBS (pH 7.4) and human serum for different times, respectively. From Fig. S3, the PBS and human serum have nearly no effect on the stability of DNA circuit during a reasonable period of time. Notably, compared with the naked siRNA, the DNA circuit presents a significantly enhanced stability either in PBS or in serum, which demonstrate the feasibility of the proposed DNA circuit in complex physiological environment.


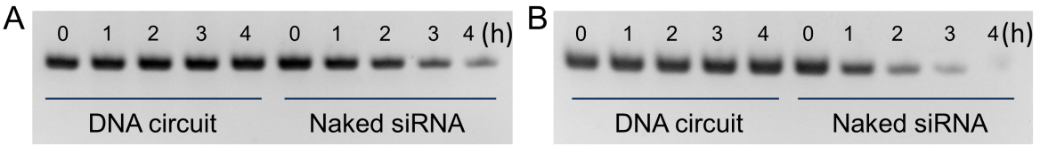


**Fig. S3.** Stability of DNA circuit and naked siRNA after incubated with PBS (A) and (B) serum for different times, respectively. The initial concentration of each DNA specie is 10 μM.

**
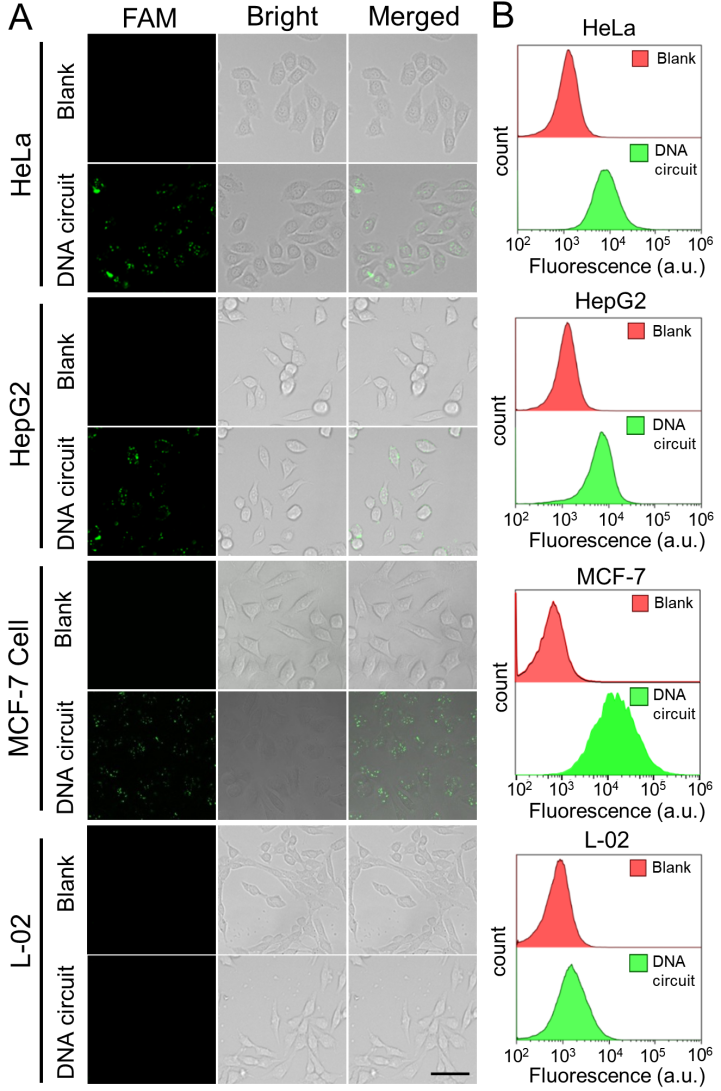
**

**Fig. S4.** MiR-21 triggered enzyme-free DNA circuit in different cells. (A) CLSM images and (B) corresponding flow cytometric results of HeLa cells, HepG2 cells, MCF-7 cells and L-02 cells after incubated with DNA circuit for 4 h. The final concentration of each DNA hairpin is 200 nM. Scale bar in A: 50 μm.


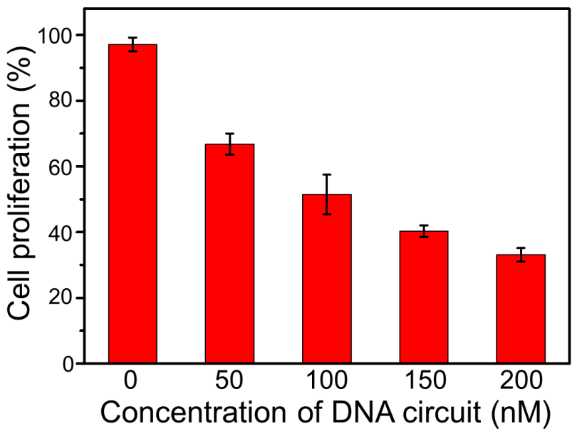


**Fig. S5.** Proliferation of HeLa cells after treated with various concentrations of DNA circuit for 48 h at 37 ^o^C. The data error bars indicate means ± SD (n = 3).


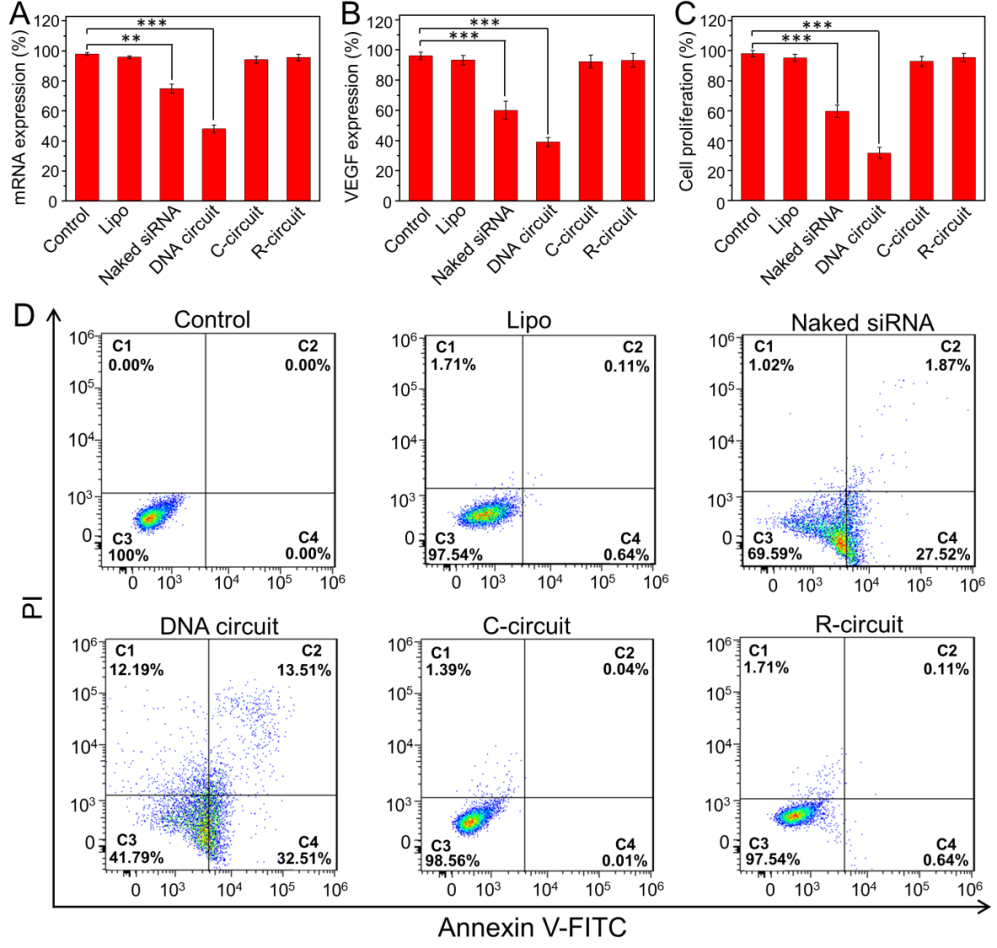


**Fig. S6.** Intracellular therapeutic effect of DNA circuit on HepG2 cells. (A) Real-time PCR characterization of VEGF mRNA expression. (B) Western blot characterization of VEGF protein expression. (C) Proliferation of HepG2 cells after different treatments for 48 h at 37 ^o^C. The final concentration of the sample is 200 nM. The HepG2 cells without any treatment are the control. (D) Apoptosis of HepG2 cells after incubated with different samples. The cell apoptosis is tested with Annexin V-FITC/PI apoptotic kit. Data error bars indicate means ± SD (n = 3). **P < 0.01, ***P < 0.001


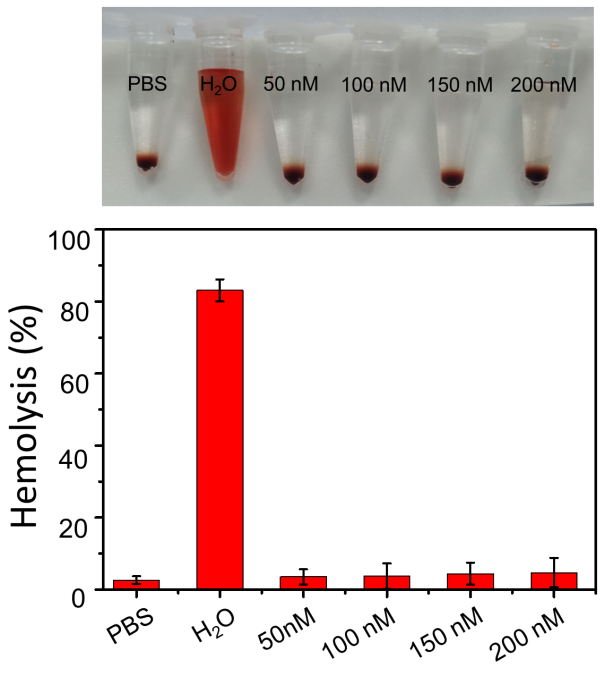


**Fig. S7.** Hemolysis assay of DNA circuit with different concentrations.

***In vivo* antitumor efficacy of liposome**

The *in vivo* antitumor efficacy of liposome is evaluated (Fig. S8). From Fig. S8a, compared with the control group, there is no obvious difference of body weight when the mice are only treated with liposome, demonstrating the nontoxicity of liposome. Moreover, from Fig. S8b the tumor volumes of both PBS and liposome treated groups increase rapidly during the treatment, showing their little inhibition on tumor growth. The average weight and corresponding photos of removed tumors in different groups are shown in Fig. S8c and Fig. S8d, respectively. Furthermore, to test the side effect of liposome, the tumor tissues are analyzed via H&E staining and TUNEL assay (Fig. S8e and Fig. S8f). As expected, the tissue and tumor slices show nearly no cell apoptosis and necrosis in lipo-treated group, demonstrating its excellent biocompatibility.


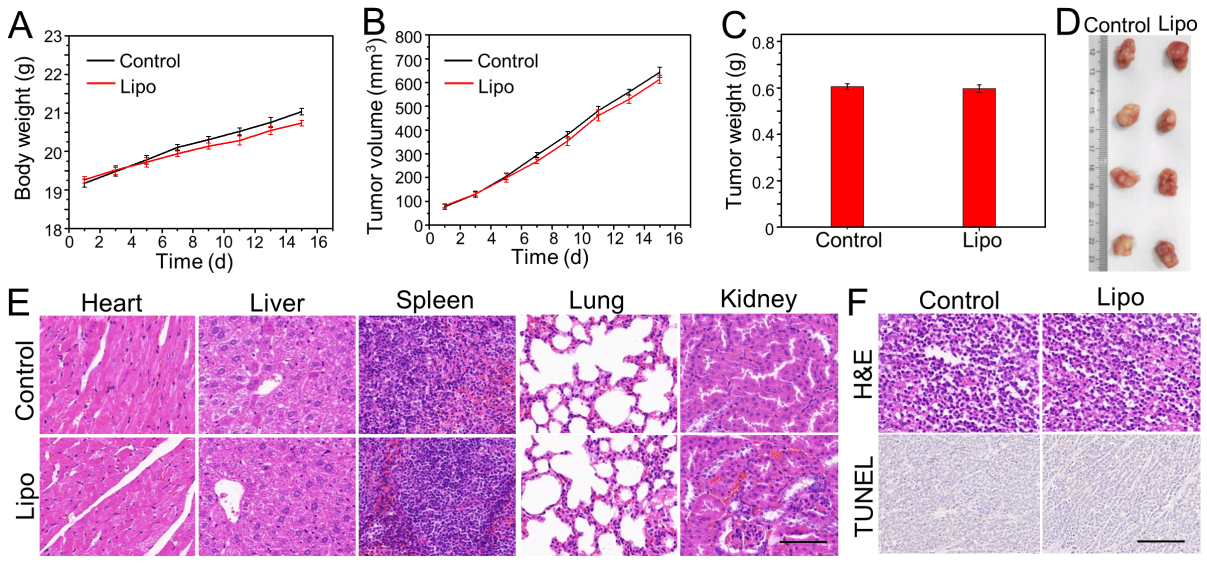


**Fig. S8.** *In vivo* antitumor efficacy of liposome. (A) Body weight and (B) tumor volume changes of mice during therapy. (C) Tumor weight and (D) corresponding photographs of tumors dissected from HeLa tumor-bearing mice at the therapeutic terminal. (E) H&E staining of tumors and major organs (heart, liver, spleen, lung and kidney) from mice with PBS and liposome treatments. (F) TUNEL staining of the tumor tissues with PBS and liposome treatments. Scale bar: 100 µm. Error bars indicate means ± SD (n = 4).


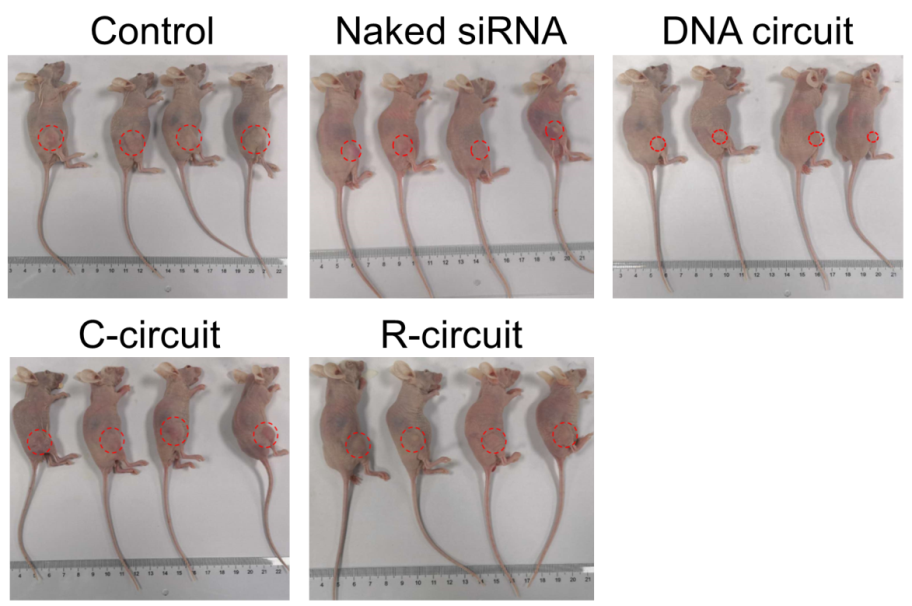


**Fig. S9.** Photographs of mice with different treatments at the therapeutic terminal.

**Table S1** Sequences of oligonucleotides used in this work

| **Name** | **Oligonucleotide Sequences (5′- 3′)** |
| --- | --- |
| miR-21 | UAGCUUAUCAGACUGAUGUUGA |
| 1mis miR-21 | UAGAUUAUCAGACUGAUGUUGA |
| 3mis miR-21 | UAUCUUAGCAGACUCAUGUUGA |
| miR-145 | GUCCAGUUUUCCCAGGAAUCCCU |
| anti-sense miR-21 | UCAACAUCAGUCUGAUAAGCUA |
| H1 | ATCAGACTGATGTTGACAGGACCGCGACGATCTCATCTCAACATCAGTCTGATAAGCTA |
| H2 | GAUCUCAUC AGGGUACUCC TAGCTT GATGAGATC GTCGCGGTCCTG TCAACAT |
| H3 | FAM-AGGGTACTCCTAGCTTATCAGACTGATGTTGAAAGCTAGGAGUACCCUGAUGAGAUC-BHQ1 |
| PS | CAGGACCGCGAC |
| naked siRNA | sense: GGAGUACCCUGAUGAGAUC  antisense: GAUCUCAUCAGGGUACUCC |
| negative control siRNA | sense: UGUAGAGAUGCGGUGGUCCUU  antisense: AAGGACCACCGCAUCUACA |
| H1-C | ATCAGACTGATGTTGACAGGACCGCGACAAGGACCACCGTCAACATCAGTCTGATAAGCTA |
| H2-C | AAGGACCACCGCAUCUCUACATAGCTTCGGUGGUCCUUGTCGCGGTCCTGTCAACAT |
| H3-C | CAUCUCUACATAGCTTATCAGACTGATGTTGAAAGCTAUGUAGAGAUG CGGUGGUCCUU |
| H1-R | ATCAGACTGATGTTGA CAGGACCGCGACGATCTCATCATCTACCGGATCAGATCCTTCG |
| H2-R | GAUCUCAUCAGGGUACUCC CGAAGG GATGAGATC GTCGCGGTCCTGATCTACC |
| H3-R | FAM-AGGGTACTCCCGAAGGATCTGATCCGGTAGATGGAGUACCCUGAUGAGAUC-BHQ1 |
| forward prime (VEGF) | AGGAGTACCCTGATGAGATCGAGTA |
| reverse prime (VEGF) | TGGTGAGGTTTGATCCGCATA |
| forward prime (GAPDH) | CATGTTCGTCATGGGTGTGAA |
| reverse prime (GAPDH) | GGCATGGACTGTGGTCATGAG |

The sequence of mistake base is highlighted in red.
